# Validity and reliability of a Chinese language suicide screening questionnaire-observer rating (CL-SSQ-OR) assessment for children/adolescents

**DOI:** 10.3389/fpsyt.2023.1147161

**Published:** 2023-05-03

**Authors:** Haiping Yu, Hongjun Tian, Tao Fang, Qiuyu Zhang, Lina Wang, Xiaoyan Ma, Ranli Li, Langlang Cheng, Chuanjun Zhuo

**Affiliations:** ^1^Department of Psychiatry, Wenzhou Seventh Peoples Hospital, Wenzhou, China; ^2^Department of Psychiatry, Tianjin Fourth Center Hospital, Nankai University Affiliated Tianjin Fourth Center Hospital, Tianjin, China; ^3^Department of Emergency Psychiatry, Tianjin Anding Hospital, Nankai University Affiliated Tianjin Anding Hospital, Tianjin Mental Health Center of Tianjin Medical University, Tianjin, China

**Keywords:** validity, reliability, suicide, CL-SSQ-OR, children, adolescents

## Abstract

**Background:**

A Suicide Screening Questionnaire-Observer Rating (SSQ-OR) has been used to assess risk of suicide among individuals and to help clinicians identify and rescue individuals attempting suicide. To prevent the risk of suicide in China, a Chinese language SSQ-OR (CL-SSQ-OR) needs to be introduced.

**Objective:**

To test the validity and reliability of a CL-SSQ-OR.

**Method:**

A total of 250 individuals were enrolled in this study. Each completed a CL-SSQ-OR assessment, Patient Health Questionnaire-9, and the Beck Scale for Suicide Ideation. Confirmatory factor analysis (CFA) was adopted to determine structural validity. Spearman correlation coefficients were adopted to determine criterion validity. An internal correlation coefficient (ICC) was used to test inter-consistency and Cronbach’s *α* coefficient was used to test split-half reliability.

**Results:**

CFA was conducted with use of the maximum variance method to evaluate the item results. All of the items received scores >0.40. In addition, good model fit indices were observed for the two-factor structure RMSEA = 0.046, TLI = 0.965, CFI = 0.977. The items’ factor loading of the CL-SSQ-OR in the first factor ranged from 0.443 to 0.878. The items’ factor loading of the CL-SSQ-OR in the second factor ranged from 0.400 to 0.810. The ICC of the total CL-SSQ-OR was 0.855. Cronbach’s *α* was 0.873.

**Conclusion:**

The CL-SSQ-OR described here demonstrates ideal psychometric properties and is found to be a suitable tool for screening Chinese children/adolescents who are at risk of suicide.

## Introduction

Nearly one million suicide deaths are reported each year worldwide, with suicide being the second leading cause of death among young people aged 15–29 years (1–7). Both suicidal thoughts and suicidal behaviors are caused by a complex reciprocal action of various factors, including nature and nurture factors. For example, suicide-related genetics, social deprivation, cyberbullying, depressive moods, and alcohol abuse are key factors. Overall, suicide rates in low-and middle-income countries (LMIC) are lower than the rates in high-income countries (HIC) (11.2 vs. 12.7 per 100,000 population, respectively). While the majority of suicide deaths occur in LMICs, there remain ongoing challenges in collecting accurate suicide figures in many countries. The World Health Organization (WHO) has published major strategic documents regarding a global approach to suicide prevention. These documents include the Global Mental Health Action Plan (2013–2020), the WHO report Preventing Suicide: A Global Imperative (2014), and the United Nations Sustainable Development Goals (SDGs) (2030). A target of reducing premature mortality due to noncommunicable diseases by one-third has also been presented, with suicide mortality rate identified as an indicator for this target. Although suicidal ideation has been linked to a higher risk of death by suicide, open discussion of suicidal thoughts and/or plans to friends and family, or seeking of professional help before attempting suicide, is not common. In China, a high suicide ideation rate has been reported, especially among adolescents. Zhang et al. reported prevalences of 15.1%, 7.2%, and 3.5% for suicide ideation, plans, and attempts, respectively, over the past year in China (4, 8, 9). Despite attempts to predict suicide risk over the past 50 years, an effective method remains to be established (10, 11). Moreover, due to lack of a useful assessment tool, suicide risk screening and suicide prevention efforts are limited. Among suicide deaths, those involving adolescents and children represent a large proportion (12–15). In particular, since adolescents and children are generally not proactive in reporting their suicide risk, their guardians often underestimate or neglect the potential suicide risk of their children. This further facilitates the risk of suicide (16). Therefore, development of an observer rating tool, and its use in combination with other self-report tools, could help screen adolescents and children by employing multiple perspectives (13–17). Over the past three years in China, the prevalence of suicide has increased. In 2021, the “China Child Suicide Report” released by the Children’s Development Center of the Medical Department of Peking University highlighted that China ranks first in the world in child suicide. Approximately 100,000 adolescents die due to suicide each year, with two deaths and eight attempts every ten minutes. According to the “China Health and Family Planning Statistical Yearbook,” suicide has become the third leading cause of death among adolescents aged 10–25 years among all non-disease causes of death. Hence, prevention of suicide among adolescents and children is an important task, and a suitable assessment tool is critical for obtaining information related to suicide.

Many suicide risk assessment tools have been established which are valuable for suicide prevention efforts. To the best of our knowledge, there are currently 38 scales available for assessing suicide risk (18). Some of these include: the Columbia Suicide Severity Rating Scale (CSSR) which assesses suicide severity (19), the Suicidality Scale (SS) which focuses on mapping the suicidal mind (20), the Ask Suicide-Screening Questions (ASQ) which surveys suicidal ideation and lifetime suicide attempts (21), the Suicidal Ideation Attributes Scale (SIDAS) which mainly focuses on assessing suicidal ideation (22), the Suicidal Behaviors Questionnaire-Revised (SBQ-R) which assesses suicidal behavior (23), and the Beck Scale of Suicide Ideation (BSSI) which describes characteristics and the severity of suicide ideation (24). However, almost all of these tools tend to focus on a particular risk factor (e.g., suicidal behavior, suicidal thoughts, etc.) (25, 26). In addition, nearly all of the above-mentioned tools are self-reporting tools and do not assess suicide-related risks from other perspectives (27–29). For assessments of patients with depression, the BSSI is more suitable. However, the latter scale is not preferred for assessing risk of suicide in individuals with other mental disorders and/or non-mental disorders (30–32). Hence, a tool which can identify individuals at high-risk for suicide, especially among adolescents, is still needed.

An observer-rated screening tool that incorporates multiple perspectives is needed to identify suicide risk for individuals, particularly for children and adolescents. Professor Young-Hwan Choi established a Suicide Screening Questionnaire-Observer Rating (SSQ-OR) (33) which provides more information for a comprehensive assessment of suicide effects in adolescents and children. Due to the cultural differences that exist between different countries, use of an assessment tool which is initially established in a particular country must be first subjected to tests of reliability and validity according to the background of the country into which it is to be applied in clinical practice. For example, the Ask Suicide-Screening Questions (ASQ) (21) was introduced into the Korean language by Kim et al. from the National Institute of Mental Health (United States), the Cluster Headache Impact Questionnaire (CHIQ) was translated into the Italian language by Onofri et al. (United States), and the Central Sensitization Inventory (CSI-9) was translated into the Chinese language by Liang et al. (United States). Moreover, all of these tools were subjected to validity and reliability tests in the country into which they were introduced (34–36). Consequently, to introduce the SSQ-OR into China, we first tested the validity and reliability of the Chinese language version of this tool. Therefore, the aim of this study was to test the validity and reliability of a Chinese language SSQ-OR (CL-SSQ-OR) for preventing suicide among adolescents and children in China.

## Materials and methods

### CL-SSQ-OR

The CL-SSQ-OR evaluated included 25 items according to a Likert scale. The score for each item ranges from 1 (very in-appropriate/not important) to 5 (very appropriate/important). Please refer to Table 1 for a list of the items surveyed.

**Table 1:** Items of the CL-SSQ-OR.

Item no.	Item text
1	Rarely meets anyone and spends most of the day alone
2	Suffers from conflicts with family members such as parents, children, or siblings
3	Suffers from conflict or breakup with a love object
4	Depreciates or regards himself/herself as pathetic
5	Impulsively does something dangerous or regrettable (e.g., drunk driving, violence, binge eating, impulsive consumption, severe argument with people around)
6	Says that he/she will die when emotions get intense
7	Has planned a place, time, method for suicide (e.g., checking out a place or buying a tool, etc.)
8	Has attempted suicide more than once to date
9	Says that death is the only way to solve the current problems
10	Talks about suicide or death
11	Suddenly organizes the surroundings (e.g., property arrangement, personal affair arrangement, efforts to improve relationships)
12	Says that people around him/her would be better off if he/she dies or disappears
13	Says that he/she wanted to die following a deceased family member, friend, pet, or celebrity
14	Looks depressed or lethargic almost every day
15	Has hurt him/herself to the extent of leaving a scar
16	Continues drinking even though drinking causes serious problems (e.g., deterioration of health, violence/abusive language, interpersonal conflicts)
17	Looks very anxious and nervous
18	Has severe mood swings
19	Is suspicious of other people’s intentions and/or thinks others are doing him/her harm
20	Complains about sleep problems (e.g., not being able to fall asleep easily, waking up in the middle of the night, change in sleeping hours)
21	Suffers from a failure (e.g., job, promotion, business, academic failure, etc.)
22	Suffers from the financial distress (e.g., debt, poverty, bankruptcy, etc.)
23	Has suffered since experiencing physical, verbal, or sexual violence
24	Suffers from being unemployed
25	Suffers from unfair treatment or insult

### Translation and optimization

Initially, we translated the SSQ-OR (33) into the Chinese language (CL-SSQ-OR). Then, we invited a native Korean language speaker (Sun Yang Kim, a medical doctor trained at Tianjin Chinese Tradition Medical University) to translate this CL-SSQ-OR back into an English-language version. The final version of the CL-SSQ-OR was acquired from the harmonized Korean language version.

### Validity evaluation

To determine structural validity, confirmatory factor analysis (CFA) was adopted. Spearman correlation coefficients were adopted to determine criterion validity for the BSSI (37) and Patient Health Questionnaire-9 (PHQ-9) (38). Both the BSSI and PHQ-9 were adopted as criteria.

### Reliability evaluation

A total of 250 participants were assessed independently by twelve raters who were blinded to their suicide status. An internal correlation coefficient (ICC) was used to test inter-consistency (39), while Cronbach’s *α* coefficient was used to test split-half reliability (40).

### Cut-off point selection

Twelve professional doctors with more than 20 years of experience in suicide crisis intervention were employed to apply a consistent clinical standard. These doctors determined the area under the receiver operating characteristic (ROC) curve (AUC) to be acceptable (41) to the subjects. They also judged the cutoff points for the severity of suicide features and calculated sensitivity and specificity for various CL-SSQ-OR scores to evaluate severity of suicide features.

### Statistical analysis

Statistical Package for Social Sciences 21.0 (SPSS Inc., Chicago, IL, United States) was used to evaluate both validity and reliability for variables and to determine cutoff scores (42). CFA was applied to test constructive validity of the CL-SSQ-OR. Relationships between SSQ-OR scores and scale of suicide ideation scores (43) were analyzed with the Spearman correlation test. The internal consistency of the scale was evaluated by calculating Cronbach’s *α* coefficient and an ICC. CFA was also used to determine structural validity. The root mean square area of approximation (RMSEA) was expected to be less than 0.05, while confirmatory fit index (CFI), goodness of fit index (GFI), and Tucker–Lewis index (TLI) values were expected to be greater than 0.95 in order to consider acceptability of the model fit by CFA. The significance value was set as *p* < 0.05, within the 95% confidence interval (95% CI) (44–49).

## Results

### Participants

Participants in this study were recruited from the Department of Psychological Consultation/Suicide Crisis Intervention Center (Tianjin Fourth Center Hospital, China) and from the Department of Psychological Consultation (Wenzhou Seventh Peoples’ Hospital, China) between September 2022 and November 2022. These individuals had visited the psychological emergency room due to a suicide crisis. Inclusion criteria for this study were: (1) age 12–17 years; (2) at least 6 years of education and an ability to understand the scale of the SSQ-OR; and (3) having at least one visit to a crisis intervention center due to suicide within the last year. Exclusion criteria were: (1) age ≥ 18 years, (2) intellectual disabilities, (3) neurodegenerative disease, (4) history of a personality disorder, (5) brain trauma, and (6) any other factors which could potentially interfere with this study. After conveying a full description of the present study, guardians of the participants signed a consent form. All of the participants were interviewed by trained psychological doctors of the Tianjin Fourth Center Hospital and Wenzhou Seventh Peoples’ Hospital. All of the participants also completed a set of self-assessment questionnaires. For the CL-SSQ-OR, needed information was provided by friends, classmates, teachers, etc. The Ethics Committee of the Tianjin Fourth Center Hospital approved this study (IRB no. TW-2022-08-190).

### Construct validity

CFA disclosed that the participants’ data were suitable for factor analysis based on Bartlett’s test of sphericity and the Kaiser–Meyer–Olkin (KMO) measure (50, 51). In this study, Bartlett’s *χ*^2^ value of 2898.74 and a KMO value of 0.87 (*p* < 0.01) met the conditions for CFA. The cumulative variance contribution rate was 82.53%. CFA was conducted with use of the maximum variance method to evaluate the item results. All of the items received scores >0.40 (Table 2). Good model fit indices were also observed for the two-factor structure using CFA (RMSEA = 0.046, TLI = 0.965, CFI = 0.977). The contribution rate of the principal factor was 63.589%, which was higher than the standard 50% value of the structural validity test. Promax rotation (52) further demonstrated that each item had a high factor load (0.428–0.885; Table 2).

**Table 2:** Factor loading of the CL-SSQ-OR.

Items	Factor loading, 95%CI	*Z*	*p*
1	0.571, 0.467–0.700	14.880	<0.001
2	0.428, 0.385–0.566	15.589	<0.001
3	0.671, 0.500–0.821	13.569	<0.001
4	0.636, 0.474–0.828	16.247	<0.001
5	0.533, 0.400–0.719	16.660	<0.001
6	0.690, 0.452–0.767	14.377	<0.001
7	0.574, 0.411–0.655	24.432	<0.001
8	0.724, 0.527–0.884	18.859	<0.001
9	0.828, 0.735–0.943	16.882	<0.001
10	0.567, 0.451–0.754	20.396	<0.001
11	0.557, 0.408–0.601	25.655	<0.001
12	0.500, 0.467–0.700	16.533	<0.001
13	0.513, 0.485–0.560	15.297	<0.001
14	0.625, 0.500–0.821	16.460	<0.001
15	0.677, 0.574–0.820	18.441	<0.001
16	0.633, 0.435–0.720	13.789	<0.001
17	0.577, 0.452–0.754	15.456	<0.001
18	0.605, 0.522–0.685	14.523	<0.001
19	0.774, 0.577–0.899	16.720	<0.001
20	0.880, 0.735–0.990	15.831	<0.001
21	0.423, 0.347–0.700	17.951	<0.001
22	0.635, 0.588–0.741	17.157	<0.001
23	0.585, 0.452–0.931	15.662	<0.001
24	0.652, 0.555–0.655	18.582	<0.001
25	0.620, 0.521–0.750	15.688	<0.001

### Criterion validity

Spearman’s rank correlation coefficient was 0.900 between the SQR-OR and the BSSI. Spearman’s rank correlation coefficient was 0.975 between the SQR-OR and the PHQ-9.

### Reliability data

The total ICC value of the inter-rater consistency was 0.853, indicating good adaptability of the scale. In addition, Cronbach’s *α* coefficient of the total scale was 0.929, indicating good split-half reliability (47–49). With a clinical evaluation standard of personality features used as a reference, the ROC indicated a cutoff score ≥ 32 which is accompanied by a sensitivity value of 0.967 and a specificity value of 0.854 (Table 3). The AUC value is 0.892. Values >32 were considered to indicate a severe suicide feature. For a cutoff score ≥26, the sensitivity value is 0.915 and the specificity value is 0.830 (Table 3). The AUC is 0.875. Values between 32 and 26 were considered to indicate a moderate suicide feature. When the cutoff score is ≥10, the sensitivity value is 0.964 and the specificity value is 0.838 (Table 3). The AUC is 0.825. Values >10 and <26 were considered to indicate a mild severity suicide feature.

**Table 3:** Cutoff scores of CL-SQR-OR.

Cutoff score	Sensitivity	Specificity	AUC, 95% CI
32	0.967	0.854	0.892, 0.755–0.913
26	0.915	0.830	0.875, 0.680–0.904
10	0.964	0.838	0.825, 0.733–0.990

## Discussion

The present data demonstrate that the CL-SSQ-OR exhibits good validity and reliability, and can be used as an assessment tool to examine suicidal features of adolescents/children. The data provided by ROC analysis indicate that the CL-SQQ-OR can further be used to evaluate the severity of suicide problems in Chinese adolescents/children.

Validity is very important for an assessment tool, with good validity providing more precise information in clinical screenings. CFA was used to confirm the construct validity of the CL-SSQ-OR. Bartlett’s *χ*^2^ value was 599.75 and the KMO value was 0.895 (*p* < 0.01). The cumulative variance contribution rate was 69.58%. These parameters support the constructive validity of the CL-SSQ-OR and its application as an assessment tool for screening suicide features of adolescents/children. The Spearman rank correlation coefficients between the CL-SSQ-OR and BSSI, and between the CL-SSQ-OR and PHQ, were 0.902 and 0.987, respectively. These data demonstrate that the CL-SSQ-OR exhibits ideal criterion validity (52–55). Furthermore, correlation of the CL-SSQ-OR with the state versus trait loneliness scale indicates that the CL-SSQ-OR can be used to assess the severity of personality features (52–55).

Reliability is also very important for a scale. Good reliability can provide more consistent information when screening individuals with specific characteristics. Our data demonstrate that the inter-rater consistency of the ICC and the split-half reliability according to Cronbach’s *α* coefficient analysis both converge to indicate ideal reliability of the CL-SSQ-OR. More notably, use of the ROC method demonstrates that the scores of the CL-SSQ-OR can discriminate mild, moderate, and severe suicide features.

### Respondents’ opinions

After completing the CL-SSQ-OR, all of the participants were asked to complete an additional survey to obtain their opinion regarding use of the CL-SSQ-OR in this study. All of the participants expressed support for the CL-SSQ-OR as a suitable tool for assessing suicide risk at our Consultation/Suicide Crisis Intervention Center.

### Limitations

There were five limitations associated with the present study. The first limitation is that the CL-SSQ-OR has the potential for systematic distortion to accompany the rater’s observations. Hence, when this tool is used, the user should complete the inconsistent training. The second limitation of this study is that use of the CL-SSQ-OR may make it difficult to differentiate among many individuals at a low level for SSQ-OR. The third limitation is that although the CL-SSQ-OR exhibits good psychometric properties in the present study, multiple sites are needed to test its validity and reliability, and also to further confirm its psychometric properties. The fourth limitation is that only adolescents who visited the crisis intervention center of our hospital were enrolled. Hence, lack of a health control (due to the stigma of mental health, healthy control adolescents are very difficult to recruit in China) is a flaw of the present study. In a future study, we will recruit healthy controls and evaluate the CL-SSQ-OR as an assessment tool for the general population of adolescents in China. Finally, the fifth limitation of the present study is that the BSSI and PHQ were both used to establish validity. However, neither the BSSI nor PHQ have been tested on cohorts which included adolescents and/or children. Clinical experience was used to define the criteria for selecting cutoff points, and the validity and reliability of these cutoff points in children and adolescents should be tested in future studies.

## Conclusion

Our data demonstrate that the CL-SSQ-OR exhibits ideal validity and reliability in screening the suicide features of children and adolescents in China, and was able to further indicate the severity of the suicide features identified. To the best of our knowledge, there are very few scales available which can be used to assess suicide features among Chinese adolescents. Therefore, the present study provides a tool which can potentially provide new insights into the suicide features of adolescents and children to improve screening and treatment.

## Data availability statement

The raw data supporting the conclusions of this article will be made available by the authors, without undue reservation.

## Ethics statement

The studies involving human participants were reviewed and approved by Ethics Committee of the Tianjin Fourth Center Hospital. Written informed consent to participate in this study was provided by the participants’ legal guardian/next of kin.

## Author contributions

HY, HT, TF, and QZ conceived and designed research. LW, XM, RL, and LC collected data and conducted research. HT and CZ analyzed and interpreted data. HY and LC wrote the initial draft. HT and XM revised the manuscript. CZ had primary responsibility for final content. All authors contributed to the article and approved the submitted version.

## Funding

This work was supported by the Wenzhou Science and Technology Bureau (Y20180563 to HY).

## Conflict of interest

The authors declare that the research was conducted in the absence of any commercial or financial relationships that could be construed as a potential conflict of interest.

## Publisher’s note

All claims expressed in this article are solely those of the authors and do not necessarily represent those of their affiliated organizations, or those of the publisher, the editors and the reviewers. Any product that may be evaluated in this article, or claim that may be made by its manufacturer, is not guaranteed or endorsed by the publisher.
